# Trauma‐informed practice for children and young people with intellectual disabilities: A scoping review

**DOI:** 10.1111/bjep.70011

**Published:** 2025-07-31

**Authors:** Claire Wilson, Zara P. Brodie, Kirsten Russell

**Affiliations:** ^1^ School of Education and Social Sciences, The University of the West of Scotland, UWS Paisley Campus Paisley UK; ^2^ School of Health in Social Science, The University of Edinburgh Edinburgh UK; ^3^ School of Psychological Sciences and Health, The University of Strathclyde Glasgow UK

**Keywords:** contexts of learning, methodological issues, registered reports, special education – atypical development, teacher education

## Abstract

**Background:**

There is growing consensus that children and young people (CYP) with an intellectual disability are more likely to experience trauma than those in the general population, which can exacerbate their vulnerabilities and developmental challenges. Trauma‐informed practices (TIPs) have been used to support those who have experienced trauma, but we need to know more about the implementation of TIPs with this population.

**Aim:**

The study aimed to synthesize research investigating TIPs for CYP with intellectual disabilities. The review examined what TIPs, policies and models have been implemented, the effectiveness of these and barriers and facilitators of trauma‐informed intervention implementation.

**Methods:**

A scoping review was conducted of quantitative and qualitative research. In total, 11 studies met the inclusion criteria. Study findings were analysed using a narrative synthesis approach.

**Findings:**

The review identified several TIPs that have been used to support CYP with intellectual disability. However, limited intervention studies were found. Teacher training was discussed as a barrier and facilitator of TIP. Training is needed to enhance staff's ability, skills, and knowledge. It was also reported that adaptations to usual TIP can enhance efficacy.

**Conclusions:**

The findings have implications for research, practice, and policy. More intervention studies are needed with this population. Policymakers must recognize the importance of teacher training and take action to provide such opportunities. We also urge practitioners to make adaptations to usual trauma therapy when working with CYP with intellectual disability. In doing so, such individuals may overcome trauma‐related barriers and achieve positive developmental outcomes.

## CHILDHOOD TRAUMA AND ADVERSITY

Adverse childhood experiences (ACEs) are potentially traumatic childhood events, including abuse, neglect, loss of a parent and household dysfunction (Felitti et al., 1998). National studies suggest that around 50% of the UK population experiences at least one ACE, while around 12% experience four or more (Bellis et al., 2015). Research indicates that adversity and associated negative outcomes act in a dose–response manner, such that the higher the adversity a person experiences, the higher the risk of poor health and social outcomes (Felitti et al., 1998). Thus, the level of exposure to traumatic experiences has a cumulative effect (Follette et al., 1996; Sacchi et al., 2020; Suliman et al., 2009). There is increasing recognition that ACEs put pressure on public health and social systems and should be critical in informing social policies and strategy (Bellis et al., 2015). In recent years, there have been calls for service systems to implement ‘trauma‐informed’ practice, including within education (Ko et al., 2008).

Drawing on both ecological systems theory (Bronfenbrenner, 1977) and attachment theory (Bowlby, 1988), childhood adversity and trauma are influential in child development, due to the brain's susceptibility to environmental experiences in the early years of life. Neuroimaging research demonstrates that ACEs are associated with differences in brain regions responsible for emotion processing, cognition, and executive functioning; particularly self‐regulation and relationship formation (Cai et al., 2023). Issues with these aspects of functionality are especially problematic in a learning context, where success is often reliant on the ability to focus, attend to information, and engage/communicate effectively (Snowman & McCown, 2012). Research involving neurotypical children shows that ACEs are associated with poorer school performance, attendance, and feelings of exclusion (Hurt et al., 2001; Luke & Coyne, 2008). These findings are valuable but may not be generalizable to all children. We need to know more about the specific needs of those with an intellectual disability and a history of trauma, given that this group is particularly vulnerable to the impact of childhood trauma (Hughes et al., 2019). The medical definition of intellectual disability refers to this as a group of neurodevelopmental conditions involving an intelligence quotient of under 70 and limitations in adaptive functioning (Lee et al., 2023; Nouwens et al., 2017). However, a strength‐based definition views intellectual disability as associated with differences in health that can improve through person‐centred learning, decision‐making, and communication (Emerson et al., 2011; Hatton & Emerson, 2015).

## INTELLECTUAL DISABILITY AND TRAUMA VULNERABILITY

Those with intellectual disability are significantly more likely to experience and be vulnerable to trauma and multiple traumas in childhood than those in the general population (Dion et al., 2018; Mason‐Roberts et al., 2018; McNally et al., 2021; Mevissen et al., 2016), and such experiences can further exacerbate their vulnerabilities and developmental challenges (Sullivan & Knutson, 2000). This exacerbation can have significant repercussions on cognitive functioning, emotional regulation and adaptive behaviour, posing additional barriers within educational environments. Educators and caregivers face unique challenges in supporting individuals with intellectual disability who have experienced trauma.

Trauma vulnerability is heightened by the characteristics of intellectual disability and the contexts in which they occur (Rittmannsberger et al., 2018). For example, those with intellectual disability experience differences in emotional regulation, cognition, and communication, meaning they have a higher dependency on third parties to undertake daily activities, which may heighten their risk of trauma (McGlivery, 2018).

Other evidence suggests a role of the environment in that CYP with intellectual disability are more likely to experience negative family relationships and neighbourhood events than peers without disabilities (Cook & Hole, 2021). Children with a disability are twice as likely to be physically, emotionally and sexually abused as children without disabilities (Reiter et al., 2007). McDonnell et al. (2019) supported these findings in an intellectual disability sample. Jones et al. (2012) found that children with intellectual disability were four times more likely to be victims of family violence than children with no disability. Scotti et al. (2012) reported that 79% of their sample (*n* = 386) comprising CYP with intellectual disability had been exposed to at least one traumatic event.

## IMPACT OF TRAUMA ON THOSE WITH INTELLECTUAL DISABILITY

It is important to consider how trauma interacts with intellectual disability. Differences in coping resources, cognitive and adaptive skills and environmental factors such as separation from parents through early institutionalization or hospital admissions and availability of supportive social networks are likely to impact the processing of traumatic events (Mevissen et al., 2016; Tomasulo & Razza, 2007). As such, those with intellectual disability may be at a higher risk of developing PTSD after a traumatic event (Mevissen & de Jongh, 2010).

Studies reveal that children with intellectual disabilities are not only more likely to encounter trauma but are also more vulnerable to the effects of trauma due to differences in how they understand, cope, and recover from stressful events (Skelly, 2020). This heightened vulnerability is linked to communication barriers, social isolation and often increased dependency on third parties, factors that together increase the risk of exposure to abuse (McNally et al., 2021; Verdugo et al., 2020).

The Complex Trauma Taskforce (2012) argued that a single traumatic event for someone with an intellectual disability can lead to disruptions in self‐regulatory capacities. Further, similar to empirical evidence of the impact of traumatic events on mental health in the general population, research has confirmed a link between trauma and mental health in those with intellectual disabilities. Wigham et al. (2011) reviewed studies that had documented links between traumatic life events and fear, anxiety, depression, self‐harm, and post‐traumatic stress disorder. In McNally et al.'s (2021) review, a relationship between the individuals' exposure to traumatic events and outwardly aggressive behaviours was also found.

For individuals with intellectual disability, trauma exposure may lead to more pronounced developmental delays. For instance, difficulties with attention and concentration can be exacerbated by trauma, making it challenging for these children to learn and retain new information, particularly within a structured and often demanding classroom environment. These compounded challenges can manifest in behaviours such as heightened anxiety, aggression or withdrawal, potentially resulting in stigmatization and exclusion within the educational setting.

Evidence suggests that even with sufficient verbal abilities, those with intellectual disabilities may find it difficult to disclose traumatic events (Kildahl et al., 2019) and may be less likely to disclose abuse (Soylu et al., 2013) than those without intellectual disabilities. Further, trauma reactions in people with intellectual disabilities may be attributed to the underlying condition or a comorbidity such as anxiety or depression (Mevissen et al., 2016). Many clinicians do not know how to detect possible symptoms of trauma in people with intellectual disabilities (Kerns et al., 2015). Assessment of the individual's needs as a result of trauma is complex, as the clinician must have expertise in intellectual disability, trauma, mental health, and how mental health presents and is communicated by those with intellectual disabilities (Kildahl, Bakken, et al., 2019; Kildahl, Helverschou, & Oddli, 2019).

## EDUCATIONAL IMPLICATIONS OF CHILDHOOD TRAUMA IN CHILDREN WITH INTELLECTUAL DISABILITY

The impact of trauma on CYP with intellectual disability has implications for their educational experiences. Trauma can affect a Student's ability to concentrate, retain information and engage in the classroom (Souers & Hall, 2016). Many CYP with intellectual disability already experience differences in the way they learn, and trauma‐related issues can lead to further disengagement from educational activities, disruptions in behaviour and diminished academic progress.

Cumulative ACEs can impair educational attainment, especially in students with intellectual disability who face additional barriers to learning (Stewart‐Tufescu et al., 2022; Vervoort‐Schel et al., 2021). These students may struggle with school attendance, focus and classroom engagement, exacerbated by both the cognitive effects of their disability and the trauma they have experienced. Teachers who are not equipped with trauma‐informed skills may misinterpret trauma‐related behavioural symptoms as mere manifestations of the intellectual disability, potentially leading to punitive disciplinary measures rather than supportive interventions (Sullivan & Knutson, 2000). Further complicating these challenges are societal stigma and a lack of specialized resources. Schools are often underprepared to address the dual needs of trauma and intellectual disability, and educational settings may lack trauma‐informed policies, leaving these students without adequate support (Morgan et al., 2015).

## TRAUMA‐INFORMED PRACTICE IN EDUCATION: STRATEGIES AND BENEFITS

TIP is an approach that recognizes the prevalence and impact of trauma on individuals and adapts support strategies accordingly, focusing on safety, empowerment and skill building (SAMHSA, 2014). This framework may be particularly useful in educational settings for students with intellectual disability who have experienced trauma, as it emphasizes creating a supportive environment that promotes trust and psychological safety (Perry, 2006).

In trauma‐informed classrooms, educators are encouraged to foster an atmosphere of predictability and consistency as these can mitigate anxiety in trauma‐exposed CYP with intellectual disability. For example, implementing structured routines, providing clear expectations and using visual schedules can create a sense of stability and control, reducing trauma‐related triggers (Bath & Seita, 2008). TIPs also involve developing individualized strategies to support emotional regulation and coping, such as teaching calming techniques, providing sensory spaces or incorporating mindfulness activities to help students manage stress and improve focus (Souers & Hall, 2016). TIP helps educators differentiate between behaviours arising from trauma and those associated with intellectual disability. Training teachers to identify trauma responses allows them to provide targeted support rather than punitive measures, which are often counterproductive for children who have experienced trauma (Ko et al., 2008).

### Building relationships and trust

Developing strong, trusting relationships with students is central to TIP. Research suggests that positive relationships with caregivers and educators can act as protective factors for those who have experienced trauma, providing a buffer against further psychological harm (Cook et al., 2005). Teachers can build trust by demonstrating consistency, patience and empathy, creating a supportive environment that helps students feel secure and valued (Craig, 2016). Such relationships are particularly important for CYP with intellectual disability who may struggle to articulate their needs (Morgan et al., 2015).

### Emotional regulation and skill building

TIPs also emphasize skill building in emotional regulation and interpersonal interactions. Given that trauma can disrupt a child's ability to manage stress and navigate social situations, educators trained in TIPs can employ techniques such as mindfulness, relaxation exercises and social skills training to help students develop healthier coping mechanisms (Steele & Malchiodi, 2012). These strategies can be particularly helpful for children who may react to trauma with heightened aggression, anxiety or withdrawal, providing them with tools to express and manage their emotions more effectively in both academic and social settings.

### Creating trauma‐informed learning environments

A trauma‐informed learning environment requires accommodations that account for both the intellectual and emotional needs of the student. This might include the use of sensory‐friendly spaces, flexible seating options and other classroom adaptations that reduce sensory overload and help students remain calm and focused (Bath & Seita, 2008). Additionally, trauma‐informed educators are encouraged to implement individualized learning plans that account for each student's unique needs, capabilities and learning style, creating an inclusive environment that leads to improved educational outcomes.

## THE CURRENT STUDY

The intersection of childhood trauma and intellectual disability presents a range of challenges that can significantly impede a child's educational experience and overall development. Understanding the unique vulnerabilities of trauma‐exposed children with intellectual disability is critical to addressing the compounding effects of trauma and cognitive limitations. TIPs offer valuable strategies for creating safe, supportive and accommodating learning environments tailored to meet the needs of these students. Through trauma‐sensitive approaches, educators can foster resilience, promote emotional regulation and support academic engagement, thereby helping children with intellectual disability to overcome trauma‐related barriers and achieve more positive developmental outcomes. However, a significant first step towards building this body of work is identifying what research has thus far been conducted on the implementation of trauma‐informed approaches with CYP with intellectual disability, and the experiences of those involved. We conducted a novel scoping review to synthesize the research evidence on the implementation of TIPs in the context of working with CYP with intellectual disability. Our research questions were:

1. What TIPs, policies and models have been implemented for CYP with intellectual disability?
2. What TIPs have been effective for CYP with intellectual disability?
3. What are the barriers and facilitators in the process of implementing TIP for CYP with intellectual disability?

The review is of significance given that this will provide guidance on how to use TIPs with CYP with intellectual disability; thus, it will have implications for future research, practice and policy.

## METHOD

The review was registered with the International Prospective Register of Systematic Reviews (PROSPERO; registration CRD42024565584) and the Preferred Reporting Items for Systematic Reviews and Meta‐Analyses extension for Scoping Reviews was followed (PRISMA‐ScR; Tricco et al., 2018).

### Search strategy

A systematic literature search was conducted within seven electronic databases (PubMed, ERIC, British Education Index, EMBASE, PsycINFO/Articles and Web of Science) using keywords relating to TIP and intellectual disability (See Appendix A for full search strategy). The search was conducted up until 13 August 2024, and no date or location restrictions were applied. To be comprehensive and ensure that no papers had been missed in the original search, we carried out citation tracking and reference checking on the papers included in the first round of screening.

### Selection criteria

Criteria for inclusion in the review included:

1. Original, published research. Commentaries, letters to the editor, book chapters, dissertations and reviews were excluded from the review.
2. Published in the English language or an English translation must be available.
3. Research conducted with participants of school age (between 4 and 18). If an article included other age groups outside of this age range, it was included only if the results were broken down by age group and available for CYP with intellectual disability age 4–18.
4. Papers including participants with intellectual disability or who work with CYP who have intellectual disability (e.g., staff).
5. Research directly examining TIP.

For the purposes of the review, intellectual disability relates to differences in cognitive functioning and adaptive behaviour. Thus, children with intellectual disability learn, understand new or complex information, and communicate with others in different ways than those without an intellectual disability (APA, 2013; Mencap, 2024; WHO, 2001). However, as previously stated, such outcomes can be improved through person‐centred care (Emerson et al., 2011; Hatton & Emerson, 2015).

There is currently no single agreed definition of what TIP is. The UK government (2022) identified TIP as a model grounded in and directed by an understanding of how trauma exposure affects service users neurologically, biologically and psychologically. This working definition emphasizes the main components of trauma‐informed care as: (a) realizing the widespread impact of trauma, (b) recognizing the signs of trauma and (c) preventing retraumatization. This approach reflects the original internationally recognized definition developed by the United States Substance Abuse and Mental Health Services Administration (SAMHSA) and will be used for the purpose of this review.

### Selection process

Duplicate studies identified by the initial search were cross‐checked and removed. All titles and abstracts were independently screened by two authors, with reference to the inclusion/exclusion criteria. Inter‐rater reliability was assessed using Cohen's kappa (κ = .624 *p* < .001). This demonstrated substantial agreement between reviewers (Landis & Gary, 1977). Consensus was reached via discussion with a third reviewer. The third reviewer blindly reviewed papers in which reviewers disagreed. A meeting was held with all reviewers to discuss and come to a consensus. Full‐text papers were then independently assessed by two authors to determine suitability for inclusion. Disagreements were discussed and resolved with a third reviewer using the same method as above.

### Data extraction

A data extraction tool was adapted from tools employed in previous reviews (Gardani et al., 2022; Russell et al., 2019; Zortea et al., 2021) to fit the specific questions of the current review. The tool was piloted to ensure that it was fit for purpose and captured relevant data from the included papers. For each paper, the following data was extracted: (a) publication characteristics (e.g., title, authors, year, aims and study design), (b) sample characteristics (e.g., who the participants were, sample size and country), (c) TIP characteristics (e.g., TIP approach and outcome measures) and (d) results (e.g., main findings, barriers and enablers to implementation). Data were extracted by one author and independently cross‐checked by a second author.

### Quality assessment

Given that we sought to include quantitative, qualitative and mixed methods studies, the Mixed Methods Appraisal Tool (MMAT; Hong et al., 2018) was used to critically appraise the eligible studies. The authors discourage the calculation of an overall score when appraising studies and instead advise providing a more detailed presentation of the ratings of each criterion to better inform the quality of the included studies (see http://mixedmethodsappraisaltoolpublic.pbworks.com/w/file/fetch/127916259/MMAT__criteria‐manual_2018‐08‐01_ENG.pdf for the appraisal tool). Sources of potential bias were considered and acknowledged by the authors when synthesizing the findings of included studies.

### Data synthesis

Given the methodological heterogeneity across studies, it was not feasible to conduct a meta‐analysis. As such, a narrative synthesis approach was taken.

## RESULTS

**FIGURE 1:**
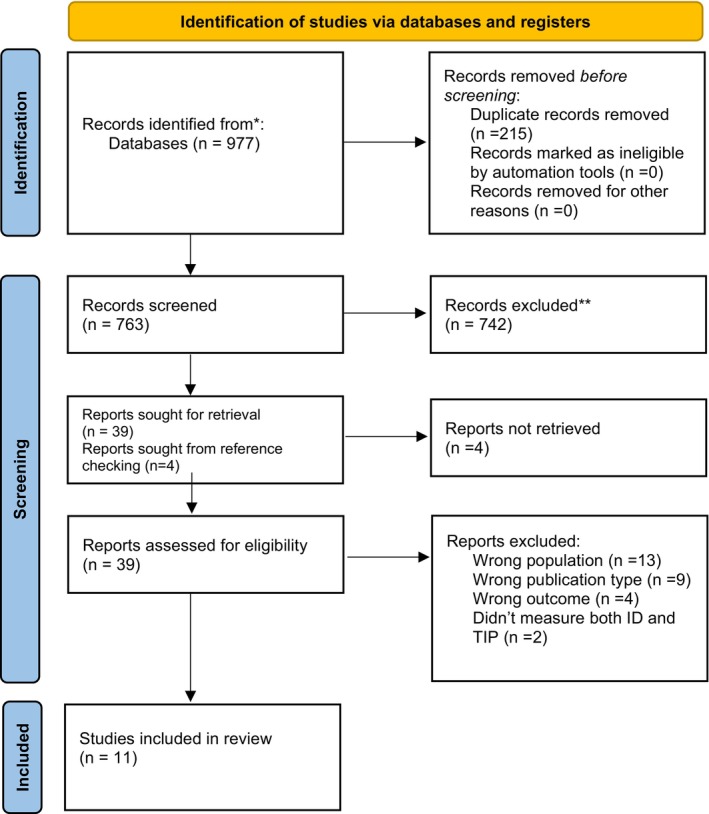
PRIMA 2020 flow diagram.

The initial search yielded a total of 977 papers. After duplicates were removed, 762 titles remained and were screened for eligibility based on their titles and abstracts. This resulted in the selection of 39 papers for full‐text assessment. In addition, four papers were identified via reference checking and citation tracking of the papers included in the full‐text assessment stage. The screening process resulted in 11 papers being included in the current review. See Figure 1.

### Study characteristics

A total of 11 studies were included in the current scoping review. Study publication dates ranged from 2013 to 2024. One study was published in 2013 (Holstead & Dalton, 2013), another was published in 2021 (Schofield et al., 2021) and the majority (*n* = 9) were published in the past 2 years. As represented in Table 1, 4 studies were qualitative (Chudzik, Corr, & Fisher, 2023; Chudzik, Corr, & Santos, 2023; Schofield et al., 2021; Southall, 2024), 2 utilized mixed methods (Chudzik, Corr, & Wolowiec‐Fisher, 2023; Gray et al., 2024), 2 implemented quantitative cross‐sectional surveys (D'Amico et al., 2021; Opoku et al., 2024), 2 used case study designs (Haydon & Kennedy‐Donica, 2023; Goldenthal et al., 2024) and one investigation conducted a quasi‐experimental study (Holstead & Dalton, 2013). Most of the work in this area of research has taken place within the United States (*n* = 9). One study was conducted in the United Arab Emirates and another in Australia.

**TABLE 1:** Study characteristics of included studies.

Author (year), country	Purpose	Research design	Sample	Main outcomes/measures used
Chudzik, Corr, and Santos (2023), USA	To explore the experience of early childhood special education professionals in one Centre to understand how their individual and collective experiences may have impacted how they planned and implemented TIC	Qualitative Collective Case Study Methodology (Interviews)	18 early childhood special education professionals including 5 administrators, 7 teachers, 3 paraprofessionals, and 3 related service providers (42.2% female)	Interview questions focused on (a) questions about their role in the school, and (b) questions about their beliefs towards TIC and experiences implementing it, including barriers and facilitators. Interview questions were designed to address multiple components of Bronfenbrenner's Ecological Systems Theory
Chudzik, Corr, and Fisher (2023), USA	To understand early childhood special education teachers' professional development needs related to trauma and trauma‐informed care.	Qualitative Study (Interviews)	18 early childhood special education teachers (100% female)	Interview questions focused on understanding (a) participants' experiences with trauma‐informed care in the classroom, (b) current knowledge of trauma‐informed care and (c) their training needs related to trauma‐informed care.
Chudzik, Corr, and Wolowiec‐Fisher (2023), USA	To explore early childhood special education teachers' attitudes and experiences with trauma and trauma‐informed practices	Mixed Methods Study (survey and interviews)	25 early childhood special education teachers (survey: *n* = 7, survey and interviews: *n* = 18)	Survey: Attitudes Related to Trauma‐Informed Care (ARTIC‐10) Scale (Baker et al., 2016) Interview questions focused on (a) teachers' definitions of trauma, (b) their understanding of intersections between trauma and development, (c) challenging behaviours they experience in the classroom, (d) experiences with trauma, trauma‐informed practices, and young children, and (e) teachers' definitions of ‘resilience’
D'Amico et al. (2021), USA	To characterize and assess the delivery of Trauma‐Focused Cognitive Behavioural Therapy with young people with Developmental Disabilities	Quantitative Cross‐sectional Study (Survey)	391 providers listed with the TF‐CBT National Registry of Certified Therapists	Survey developed to assess (a) clinician level of experience, (b) clinician comfort in working with youth with developmental disabilities, (c) strategies/adaptions utilized, (d) implementation and organizational experienced, (e) lessons learned, and (f) resources utilized to address the diverse needs of this vulnerable population
Goldenthal et al. (2024), USA	To explore the design and implementation of a three‐tiered model of trauma‐informed care in a special education setting	Case Study	22 members of clinical staff (*n* = 7) and teaching staff (*n* = 15) from a cooperative that provides services to students within a geographic area spanning 12 communities in the suburbs of a large metropolitan area	Planned data collection for Tier 1 included information regarding staff attitudes (ARTIC‐10 (Baker et al., 2016)), professional quality of life behaviours (PROQOL, Stamm (2010)) and an environmental scan checklist of trauma‐informed practices Planned data collection for Tier 2 included group implementation outcomes (i.e., the number of groups implemented and the number of students attending each), group fidelity, knowledge gained from training, and satisfaction with training
Gray et al. (2024), USA	To (1) describe the continuum of trauma‐informed services for special education students in the district, (2) describe the mental health needs and substance use of a specific population of students in special education at a large urban school district receiving intervention services, and (3) describe the adaptation process and feedback from school social workers of implementing a resilience curriculum (FRC) for special education students with emotional and behavioural difficulties as part of trauma‐informed services provided to this population.	Mixed Methods Study (survey and interviews)	814 elementary and high school students (37.6% female) and 7 school social workers.	Quantitative outcomes and measures (school students): Wellness Check Up (an online multi‐instrument screening tool for mental health, physical health, and substance use). Outcomes included PTSD (CPSS; Foa et al., 2001), depression (PHQ‐2; Arrieta et al., 2017), anxiety (GAD‐2; Spitzer et al., 2006), substance abuse (CRAFFT; Knight et al., 2002) and violent exposure (Violence Exposure Checklist) Qualitative outcomes (school social workers): interview questions focused on (a) classroom/group size and structure, (b) specific adaptations made to the curriculum, (c) challenges and successes encountered, (d) student and family response to the curriculum, and (e) recommendation they would make to future providers
Holstead and Dalton (2013), USA	To examine whether Trauma‐Focused Cognitive Behavioural Therapy is an effective treatment for those with complex trauma and co‐existing mild cognitive disabilities.	A Quasi‐Experimental Study	87 children referred to residential treatment services who had co‐existing cognitive disabilities and trauma (18% female) Participants were assigned to one of two groups. Group A (Treatment Group) were children referred by the juvenile justice system who were required by the state to be provided TF‐CBT or other trauma‐focused empirically based treatment services (*n* = 42). Group B (Control Group) were children referred by other funding sources like the Department of Education or the Bureau of Developmental Disabilities, and not required by contract to receive TF‐CBT (*n* = 45)	Pre‐ and post‐intervention measure: Internalizing and externalizing behaviours (somatic, withdrawn, social problems, anxious/depressed, thought problems, attention problems, aggressive behaviour and rule breaking) (Teacher Report Form of the Achenbach System of Empirically Based Assessment; Achenbach (2001)) Children's school teachers completed the measure
Haydon and Kennedy‐Donica (2023), USA	To examine the implementation of Acceptance and Commitment Therapy and Check‐In/Check Out with an adolescent student with a learning disability who has academic, behavioural, and intensive mental health needs	Case Study	18‐year‐old white male with a learning disability	Ability to interdependently process the traumatic distress as well as implement effective strategies to foster and sustain emotional regulation Outcome measures implemented are unclear in this study
Opoku et al. (2024), UAE	To understand the perceived competence of teachers in adopting trauma‐informed practices (TIP) for students with disabilities in regular classrooms in the United Arab Emirates	Quantitative Cross‐sectional Study (Survey)	224 classroom teachers from schools in the Emirate of Abu Dhabi	The survey collected information on the background of the teachers, Perceived Teacher Competence in term of Trauma‐Informed practice (Teacher Trauma Management Scale; Opoku et al., 2024) and Social Desirability (BIDR‐16; Hart et al., 2015)
Schofield et al. (2021), USA	To identify parent and professional perspectives on behavioural challenges experienced by young people with Prader‐Willi Syndrome (PWS) and strategies for supports, to inform understanding of how they are aligned with the TIP framework	Qualitative Study (Interviews)	14 parents of children with PWS aged between 12 and 21 years and professionals who were experienced in supporting young people with PW (3 paediatricians, one psychiatrist, and two teachers)	Interview questions focused on behaviours in the home and school environment and experience of support strategies.
Southall (2024), Australia	To explore the experiences of six teachers in regional Victoria, Australia, who sought to apply their trauma‐informed understandings after a series of professional development sessions on trauma‐informed education	Qualitative Study (Online journal, reflective circle's discussions and interviews)	6 teachers from a specialist school where students aged between 5 and 18 years older meet the criteria for a mild intellectual disability	Reflective circle discussions, online journals (completed prior to reflective circles) and interviews focused on understanding (a) the challenges that teachers experience as they implement their trauma‐informed understandings and (b) the challenges that teachers see in the transition to creating a trauma sensitive classroom

In terms of samples, the studies included in this review largely recruited teachers (*n* = 6; Chudzik, Corr, & Santos, 2023; Chudzik, Corr, & Fisher, 2023; Chudzik, Corr, & Wolowiec‐Fisher, 2023; Goldenthal et al., 2024; Opoku et al., 2024; Southall, 2024, most of whom worked within special education settings (although one investigation captured insights from those working in mainstream classrooms in the UAE)). While 4 of these studies recruited teachers only, others also included other special education professionals (i.e., administrators and related service providers; Chudzik, Corr, & Santos, 2023) and clinical staff (Goldenthal et al., 2024). It should be noted that the findings presented in these studies were not separated by participant group and thus must be considered as a whole. Sample size, participant age and where relevant, length of service information and type of trauma experienced by participants are included in Table 1.

Of those studies conducted in an educational setting, the main aims of the research were to (a) explore teachers' experiences of implementing TIC, (b) understand their attitudes towards trauma and TIP and (c) examine their professional development needs and perceived competence related to TIP. For studies conducted within a clinical context, two studies focused on investigating the delivery of Trauma‐Focused Cognitive Behavioural Therapy (TF‐CBT) with young people who have disabilities (D'Amico et al., 2021; Holstead & Dalton, 2013). One study implemented a cross‐sectional survey with certified TF‐CBT providers and sought to capture insights into their experiences of delivering TF‐CBT with CYP with Developmental Disabilities (D'Amico et al., 2021). The other study set out to examine whether TF‐CBT is an effective treatment for those with complex trauma and co‐existing mild cognitive disabilities and captured data from CYP on internalizing and externalizing behaviours pre‐ and post‐intervention (Holstead & Dalton, 2013).

One study recruited parents of children with Prader‐Willi Syndrome (PWS) and professionals who were supporting young people with PWS (i.e., paediatricians, a psychiatrist and teachers). This study sought to identify parent and professional perspectives on behavioural challenges experienced by young people with Prader‐Willi Syndrome (PWS) and strategies for support to inform understanding of how they are aligned with the trauma‐informed practice framework.

Finally, one investigation used a case study design to examine the implementation of Acceptance and Commitment Therapy and a Trauma‐Informed Check‐In/Check Out process with an adolescent student with a learning disability who also had academic, behavioural and intensive mental health needs. See Table 2.

**TABLE 2:** Intervention characteristics and main findings of included studies.

Author (year)	Intervention description	Setting	Interventionists	Main findings
Chudzik, Corr, and Santos (2023)	Service delivery that integrates an understanding of the pervasive biological, psychological, and social outcomes of trauma with the aim of ameliorating, rather than exacerbating their effects (Harris & Fallot, 2006; SAMHSA, 2014). An approach to structuring the organizational culture, practices, and policies that are sensitive to and centre the experiences and needs of individuals who have experienced trauma (McInerney & McKlindon, 2015)	Early Childhood Special Education Centre	Early Childhood Special Education Professionals.	Early Childhood Special Education professionals are impacted by factors at the micro‐, meso‐, exo‐, and macrosystem level of the environment which influenced the extent to which they were able to successfully implement TIC Participants roles in the school and training influenced their perceptions and experiences with TIC The need for explicit training on TIC to support children with disabilities was highlighted.
Chudzik, Corr, and Santos (2023)	Definition is in line with	Early Childhood Special Education	Early Childhood Special Education Teachers.	While ECSE teachers are familiar with the basics of trauma‐informed care, more training is needed, to support the successful implementation of TIC, specifically in the areas of working with families, implementing trauma‐informed classroom strategies, and supporting children with disabilities who have experienced trauma Barriers to implementation of TIC were also highlighted. These included a lack of resource (in terms of staff) and inconsistency in viewpoints of TIC across staff (i.e., some staff did not agree or understand strategies associated with TIC)
Chudzik et al. (2023)	TIC is underpinned by four key assumptions: realization of the impact of trauma; recognize the signs and symptoms of trauma; respond by fully integrating knowledge about trauma into policies, procedures, and practices; and actively resist re‐traumatization (SAMHSA, 2014)	Early Childhood Special Education	Early Childhood Special Education Teachers.	Participants' attitudes measured by the ARTIC scale did not always align with the experiences shared in the interviews. While measuring attitudes is important, changing attitudes alone may not be enough to implement change in behaviour. Early Childhood Special Education teachers need more support to successfully implement TIC in the classroom. Teachers need opportunities to learn about what trauma‐informed practices are, how to implement them in the classroom, and how to reflect on their own attitudes to further understand how they impact their teaching practices.
D'Amico et al. (2021)	Trauma‐Focused Cognitive Behavioural Therapy (TF‐CBT) Generally administered as an individually administered intervention and typically lasts between about 8 and 20 sessions. Treatment may be longer for those with co‐occurring difficulties (i.e., those with complex trauma). After the initial assessment the treatment is divided into components fitting the acronym PRACTICE, which can be divided into three phases of (1) coping, stabilization and skills building, (2) trauma narration and processing, and (3) consolidation and closure. In addition to traditional CBT components, TF‐CBT has a significant parenting skills component. Enhancing safety skills is addressed and gradual exposure is threaded throughout the treatment.	Clinical	Certified TF‐CBT therapists.	Of the participants, 34% reported comfort treating youth with developmental disabilities (DD). There was an association between experience with DD and comfort level doing TF‐CBT with this population. Discomfort varied by DD subtype Respondents reported employing a variety of flexible adaptations in session treatment time (e.g., shortening sessions, lengthening course of treatment), treatment content (e.g., simplifying language and/or using visual aids), structure, and increasing caregiver involvement Those with (some to extensive) experience treating youth with DD were more likely to report making an adjustment/flexible implementation Need for additional training and resources for increasing self‐efficacy around TF‐CBT treatment provision for youth with DD.
Goldenthal et al. (2024)	Three‐tiered continuum of support from least to most intensive Tier 1 (primary or universal) is expressly preventative and affects all children in the school through systems wide programming (e.g., instructional methods taught classroom wide). Included 4 60‐minute trauma awareness trainings designed to increase staff knowledge regarding TIC. Clinical staff received ‘Trauma in Schools’ training Tier 2 (secondary or targeted) is largely geared towards those with increased needs that do not meet clinical levels (e.g., small group interventions). Training (and supported implementation) provided to clinical staff and administration on two evidence‐based interventions – Anger Coping and Think First Tier 3 (tertiary or individualized supports) targets children at the highest level of need. Clinical staff were provided with ‘clinical challenges’ trainings that included opportunities trauma‐informed practices into school‐based supported for students with complex mental health needs.	Special Education School	Teaching and Clinical Staff	No significant changes on attitudes towards TIC or professional quality of life following Tier 1 trainings. Effort to build a more trauma‐informed environment within a special education saw some successes With regard to the Tier 2, three anger management groups were implemented and both group fidelity and student attendance was high (88%). The implementation and training of these groups was highlighted as one of the greatest successes of the partnership. Knowledge was gained through the training and staff reported moderately high levels of satisfaction with the training and supported implementation Overall satisfaction with trainings at all three tiers was moderately high. Both administration and staff described the value of the partnership in strengthening their multi‐tiered systems of support and TIC using evidence‐based practices Although the implementation of specific tiered supports demonstrated good feasibility, acceptability, and preliminary outcomes, overall, systemic issues impeded the effectiveness of the implementation of a multi‐tiered model for trauma‐informed care. Specifically, characteristics unique to special education cooperatives (e.g., makeup of multiple districts, frequent staff turnover, high levels of paraprofessionals, lack of assessment and progress monitoring tools, competing demands and limited resources) require a renewed research and policy focus including the investment of resources to fully support students in these settings.
Gray et al. (2024)	Families OverComing Under Stress (FOCUS) Resilience Curriculum (FRC), a trauma‐informed, resilience building curriculum delivered as a preventative intervention in schools. Co‐adapted to address the needs students with significant emotional and behavioural difficulties receiving special education services Delivered to elementary, middle, and high school students. Utilizes a trauma‐informed framework through psychoeducation about trauma and loss and teaching students' skills to cope with and mitigate risk of trauma exposure, while avoiding retraumatization. One of the core components of FRC is resilience skill building, with the goal of alleviating psychological distress related to traumatic events (e.g., emotion regulation, communication, problem‐solving, goal‐setting, and managing trauma stress and loss reminders) adapted from evidence‐ based interventions. Composed of 9 modules	Special Education Schools	School Social Workers	Students reported significant risk for traumatic stress, anxiety and depressive symptoms and high rates of violence exposure. Thid demonstrates the need for continued promotion of mental health awareness and development of relevant school‐based intervention programmes for students in Special Education School social workers identified numerous adaptations that can be helpful when delivering the FRC to students receiving special education services Utilizing social stories (an actual story created to help teach the skills relevant to that specific situation—for example, coping. skills/empathy) Building/strengthening coping skills (i.e., providing more opportunities to students to allow them to build the skills needed) Importance of praise/reinforcement A need to bolster communication skills (e.g., via role play) Visual reminders of skills in classrooms More involvement of teachers More communication with community therapists and other collaborating services Global considerations for adaption for this population were also highlighted by social workers: Simplifying the delivery of content Decrease session length Increased consultation between social workers so not working in isolation Continuously adapting to fit population need Providing time and space for collaborative feedback and discussion among social workers.
Holstead and Dalton (2013)	Trauma‐Focused Cognitive Behavioural Therapy (TF‐CBT) Generally administered as an individually administered intervention and typically lasts between about 8 and 20 sessions. Treatment may be longer for those with co‐occurring difficulties (i.e., those with complex trauma). After the initial assessment the treatment is divided into components fitting the acronym PRACTICE, which can be divided into three phases of (1) coping, stabilization and skills building, (2) trauma narration and processing, and (3) consolidation and closure. In addition to traditional CBT components, TF‐CBT has a significant parenting skills component. Enhancing safety skills is addressed and gradual exposure is	A private non‐profit multi‐site and multi‐service agency located. The agency serves approximately 200 children in various residential care settings and specializes in treatment services for youth who have multiple and severe problems, including developmental and severe behavioural/emotional disorders	Masters‐Level Behavioural Therapist Group A (treatment group) received 3 sessions of TF‐CBT per week. During these sessions Applied Behavioural Analysis/Individualized Behavioural Interventions (ABA/IPI) services were provided as necessary, but behaviourally based interventions were not the focus of the sessions as the TF‐CBT was used Group B (control group) received three sessions per week of APA/IBI interventions.	Manualized TF‐CBT protocol in conjunction with limited APA/IBI was not as effective as ABA/IBI services for children with cognitive and developmental disabilities. The authors suggest that this not surprising as children with cognitive and developmental disabilities pose additional challenges to treating trauma and youth who have experienced complex trauma may have difficulty with tasks associated with TF‐CBT. This issue is further exacerbated if youth have comorbid complex trauma and intellectual disabilities. Therefore ABA/IBI is more effective at treating trauma‐related symptoms in this population.
Haydon and Kennedy‐Donica (2023)	Acceptance and Commitment Therapy (strategies to modify and control the content of ones thoughts are replaced with creating more awareness and acceptance of these thoughts). The aim of ACT is not to change the frequency of unwanted private events, but to modify avoidance strategies and teach the person to align their thoughts with their values and be committed to the values through action (Luciano et al., 2012). As a result, there is a reduction of the power of unwanted thoughts on the individual Trauma informed Check In/Checkout (Tier 2 targeted intervention for students who exhibit some problem behaviour in schools). Students are student is paired with a trusted mentor teacher/staff in the school building. The student checks in at the start of each school day, mid‐day, and at the end of the day to determine how well the student is meeting school expectations or personal goals. Progress is collected on a data sheet and reinforcement, encouragement, and feedback is provided if a predetermined goal on the behaviour is met. Features of Check In/Check Out can be altered or intensified via the inclusion of conversations and elements designed to support youth with trauma. In particular, the typical Check In/Check Out structure can be slightly modified to align with the core elements of TIC—trustworthiness, felt‐safety, choice, collaboration and empowerment.	Mental Health Services/School Behaviour Team	Team‐based approach: ACT trained therapist (delivering ACT) School staff (implementing Check In/Check Out)	The combination of trauma‐informed behavioural strategies, ACT and Check In/Check Out procedures resulted in Jimmy's improved ability to interdependently process the traumatic distress as well as implement effective strategies to foster and sustain emotional regulation. These skills ultimately enabled him to achieve personal goals and take actions that aligned with these goals and values. While generalizations to large‐scale practice cannot be made from one case study, Jimmy's supports and outcomes highlight some key takeaways. In particular, a key element of Jimmy's school supports was the implementation of a package of trauma‐informed interventions and a team‐based approach.
Opoku et al. (2024)	Guided by SAMHSA's trauma model, which was designed to guide trauma‐informed practice (TIP) in classrooms and beyond. This framework consists of six components: (1) safety, (2) trustworthiness and transparency, (3) peer support, (4) collaboration and mutuality, (5) empowerment, voice and choice and (6) cultural historical and gender.	Mainstream Schools	Classroom Teachers	There was an interrelation between the domains of SAMHSA trauma management model 1: Link between background variables and TIP by teachers to support those with disabilities: Teachers in public schools were more knowledgeable than their counterparts in private schools. Teachers who indicated taking training in trauma management seemed to have better knowledge than those who indicated otherwise. Those who indicated that they were familiar with trauma policy were more knowledgeable than the others. Younger teachers seemed more knowledgeable about TIP than older teachers. High school teachers seemed more knowledgeable about trauma management than early years teachers. Classroom teachers appear more knowledgeable than especially allied professionals. However, they did not differ from special education teachers 2. Predictors of TIP by teachers who support those with disabilities: Trauma management training was the strongest predictor of TIP. Social desirable responding was also a predictor of TIP 3. Moderation effect of background variables on the relationship between social desirability and TIP: Only gender moderated the relationship between TIP and social desirability.
Schofield et al. (2021)	Guided by SAMHSA Trauma‐Informed Principles (TIP): (1) Creating a collaborative environment, (2) Empowerment, voice, and choice, (3) Trustworthiness and transparency and (4) Ensure a safe environment.	Home, education, and health	Parents, teachers, paediatricians, and psychiatrists	Strategies to support young people with PWS could be mapped onto four overarching TIP principles, in combination with three additional other domains (behavioural underpinnings, modify the environment, and family capacity building) Creating a collaborative environment emphasizes the importance of partnering with all individuals involved in care and support to increase understanding of people who have experienced trauma The Principle of Empowerment, voice and choice describes the recognition of the individual's strengths which can be built upon by the young person with PWS and those in a caregiving role Trustworthiness and transparency recognizes that all actions should be conducted with transparency with the primary goal of increasing trust between those providing support and the individual. Understanding experiences of the individual is expressed in the literature as the experiences which have brought the individual to where they are (Lisitski, 2019). Enabling elements of this in a PWS context emerged from the data as phenotype awareness Ensuring a safe environment encompasses the creation of a physically and psychologically safe environment for the young person within the home and school environments (importance of relationships, routine, and support) The inclusion of novel domains (behavioural underpinnings, modify the environment, and family capacity building) reflected the additional complexities in PWS and illustrated how to support these individuals.
Southall (2024)	Teachers had attended a series of professional development sessions run by the Department of Human Services. These sessions included the impact of extreme fear on early brain development and an overview of attachment theory. The whole staff also attended a full school closure day on trauma and its impact during early childhood, facilitated by a Department of Education team of psychologists and social workers. The teachers were then expected to implement their trauma‐informed understandings for an individual student from a traumatized background. As teachers were working within a specialized school setting an individualized approach to implementation was preferred.	Special Education Schools	Special Education Teachers.	While this group of teachers has been provided with extensive professional development about the impact of early traumatic experiences on learning, it became evident that there were many challenges that were not alleviated through understanding alone The key themes with challenges for teachers were as follows: Trauma is experienced individually Complexity of understanding the impact of trauma for an individual student. Complexity of interpreting the presenting behaviour for impact of trauma in specific contexts. Complexity of designing individually appropriate responses. Problem behaviours of students with complex trauma are repetitive Manage the exhaustion it causes. Deal with perceived feeling of being judged by peers and parents. Manage apparent loss of personal efficacy. Manage the constant rumination which ensues. Problem behaviours of students with complex trauma include aggression and avoidance To remain emotionally neutral in the face of strong personal reaction and emotion in order to build trust. Define their own personal boundaries for their role. Manage high levels of frustration, guilt, defeat and helplessness experienced. Students with complex trauma display controlling behaviours Understand the purpose of the controlling behaviour for the individual and respond in a way which promotes relationship and agency.

### What trauma‐informed practices, policies and models have been implemented to support CYP with intellectual disability?

Several approaches have been implemented to support CYP with intellectual disability who have experienced trauma. These comprised the use of a trauma theoretical framework to guide research design; the use of therapeutic interventions; and programmes addressing the school curriculum. Specifically, five studies were guided by The Substance Abuse and Mental Health Services Administration Trauma‐Informed Care Framework (SAMHSA, 2014). It should be noted that these papers used the SAMHSA (2014) framework to develop interview schedules and/or questionnaires only, and no intervention was reported. Two papers used a trauma‐focused cognitive behavioural therapy (TF‐CBT) approach, with one paper implementing an intervention for CYP with intellectual disability and one paper examining therapists' experiences of using TF‐CBT. One paper implemented an intervention for a young person with intellectual disability based on acceptance commitment therapy (ACT) and check in/check out. One paper implemented a tiered approach intervention for clinicians and teachers. One paper used a trauma‐informed resilience building school curriculum, and one paper involved teacher training on the impact of fear on children's brain development, with an overview of attachment theory also discussed. These practices and approaches are outlined below.

#### Trauma‐informed care

Five papers (Chudzik, Corr, & Fisher, 2023; Chudzik, Corr, & Santos, 2023; Chudzik, Corr, & Wolowiec‐Fisher, 2023; Opoku et al., 2024; Schofield et al., 2021) discussed The Substance Abuse and Mental Health Services Administration Trauma‐Informed Care Framework (SAMHSA, 2014). The manual provides a definition of trauma and a trauma‐informed approach offering key assumptions and principles. It should be noted, though, that this was conceptualized in different ways within these studies. For example, Chudzik, Corr, and Santos (2023); Chudzik, Corr, and Fisher (2023); Chudzik, Corr, and Wolowiec‐Fisher (2023) described the four key assumptions of the framework: (1) realizing the widespread impact of trauma, (2) recognizing the signs of trauma, (3) responding by integrating trauma‐informed care and (4) resisting retraumatization.

Opoku et al. (2024) considered SAMHSA's (2014) six key principles; (1) Safety; (2) Trustworthiness and Transparency; (3) Peer Support; (4) Collaboration and Mutuality; (5) Empowerment, Voice and Change; (6) Cultural, Historical and Gender Issues. ‘Safety’ relates to teachers creating a safe environment in which the individual feels supported to learn. ‘Trustworthiness and Transparency’ concerns fair and transparent decision‐making processes. The individual should feel listened to and empowered to have a voice. ‘Peer support’ refers to the support the individual receives from their peers. ‘Collaboration and Mutuality’ highlights the importance of collaboration between the teacher and the individual to allow the individual to receive appropriate education. Teacher and the student work together to avoid issues related to power and foster good well‐being. ‘Empowerment, Voice and Choice’ concerns the need for teachers to support the individual to be empowered to take ownership of their lives to increase positive well‐being and help them reach their potential. Finally, ‘Cultural, Historical and Gender’ guides policy formation which should discourage stigmatization or discrimination against the individual. Schofield et al. (2021) study opted to focus on four of these principles; (1) Creating a collaborative environment; (2) Empowerment voice and choice; (3) Trustworthiness and transparency and (4) Ensuring a safe environment.

#### TF‐CBT

Two papers (D'Amico et al., 2021; Holstead & Dalton, 2013) discussed TF‐CBT developed by Cohen et al. (2006, 2017). In D'Amico et al.'s (2021) study, therapists' experiences of using TF‐CBT were examined. TF‐CBT was conceptualized as comprising three phases of (1) coping, stabilization and skill building, (2) trauma narration and processing and (3) consolidation and closure. Holstead and Dalton (2013) carried out a TF‐CBT intervention for CYP with intellectual disabilities. Within this study, TF‐CBT provided post‐traumatic stress disorder psychoeducation, stress‐management skills, information relating to the cognitive triad (relationships between thoughts, feelings and behaviours), safety skills, information about healthy sexuality, the creation of a trauma narrative and desensitization. Parent training was also included. TF‐CBT, therefore, aims to address symptoms of depression and/or anxiety as well as addressing negative thoughts around self‐blame, safety and dependency on others. It is hoped that the child will develop coping skills and ways to restructure maladaptive thoughts (Allen & Johnson, 2012).

#### ACT and check in/out

Haydon and Kennedy‐Donica (2023) implemented ACT (Hayes, 2016) and Check In/Out. ACT aims to create awareness and acceptance of one's thoughts rather than replace these with strategies to control and change cognitions (Hayes, 2016). Thus, ACT is not about modifying the frequency of thinking about traumatic events but instead changing avoidance strategies and helping the individual align their thoughts with their values (Luciano et al., 2012). This then reduces the power of the negative thoughts. In addition, Haydon and Kennedy‐Donica (2023) described the Check In/Out intervention. This involves pairing the student with a trusted member of school staff who they then check in with at the start, middle and end of the day (Hawken et al., 2020). This provides insight into whether the student is meeting school expectations or their own personal targets.

#### Tiered approach

Goldenthal et al. (2024) discussed the three‐tiered continuum of support which acknowledges the importance of the severity of needs (Chafouleas et al., 2016). Tier I refers to a preventative programme aimed at all children within the school. It is argued that within this Tier, the school climate should be transformed to become trauma aware. This can be done through school strategies that highlight the role of trauma in teaching and learning. Examples of strategies given were teacher training opportunities and interventions to enhance children's feelings of safety. Tier II relates to a targeted small group intervention which is aimed at those with increased needs that do not reach clinical level. Examples included school‐based interventions to address traumatic exposure. Tier III concerns individual support and is for children at the highest level of need. This often involves referring the child to trauma services out with the school.

#### Trauma‐informed resilience building curriculum

Gray et al. (2024) discussed the implementations of Families Over Coming Under Stress (FOCUS) Resilience Curriculum (FRC). This was a trauma‐informed, resilience‐building school curriculum developed through a community‐academic partnership and delivered as a preventive intervention within schools. FRC aimed to teach the children how to cope with and minimize the risk of trauma exposure. Resilience skill building was key to this intervention with the aim of reducing psychological distress resulting from traumatic events. Resilience skills training included a focus on emotion regulation, communication, problem‐solving, goal‐setting and managing trauma, stress and loss.

#### Impact of fear on brain development with an overview of attachment theory

Southall (2024) discussed school staff training which involved sessions on the relationship between extreme fear and early brain development as well as an overview of attachment theory. Staff also attended a training day on trauma and its impact during childhood. After receiving this training, it was expected that teachers would implement their knowledge of trauma for any student from a traumatic background.

### How effective are the approaches to trauma‐informed care that have been implemented to support young people with ID?

As highlighted, most studies focused on (a) understanding teachers' experiences of implementing TIP (Chudzik, Corr, & Santos, 2023; Opoku et al., 2024; Southall, 2024) and their attitudes (Chudzik, Corr, & Wolowiec‐Fisher, 2023), (b) student experiences of receiving TIP (Gray et al., 2024; Schofield et al., 2021) and (c) examining their professional development needs and perceived competence related to TIP (Chudzik, Corr, & Fisher, 2023; D'Amico et al., 2021). Only three studies investigated the effectiveness of a trauma‐informed intervention (Goldenthal et al., 2024; Haydon & Kennedy‐Donica, 2023; Holstead & Dalton, 2013), limiting the ability to form conclusions. These studies will now be discussed.

Within a special education setting, Goldenthal et al. (2024) sought to examine the design and implementation of a three‐tiered model of trauma‐informed care (involving both teachers and clinical staff). Teir 1 included 4 60‐minute trauma awareness trainings designed to increase staff knowledge regarding TIP, and clinical staff received ‘trauma in schools’ training. Tier 2 involved training (and supported implementation) provided to clinical staff and school administration on two evidence‐based interventions, to be delivered to young people in the school. These interventions were Anger Coping and Think First. Tier 3 was aimed at children at the highest level of need and required clinical staff to attend a series of psychiatric ‘clinical challenges’ trainings that focused on case consultation didactics and opportunities to discuss opportunities to integrate TIP into school‐based support for students with complex mental health needs. No significant changes in attitudes towards TIP or professional quality of life following Tier 1 trainings. With regards to Tier 2, three anger management groups were implemented and both group fidelity and student attendance was high (88%). Overall satisfaction with training at all three tiers was moderately high. Both administration and staff described the value of the partnership in strengthening their multi‐tiered systems of support and using evidence‐based TIP.

Holstead and Dalton (2013) examined the effectiveness of TF‐CBT for children with complex trauma and co‐existing mild cognitive disability. The treatment group (Group A; *n* = 42) were provided with three TF‐CBT sessions a week, whereas the control group (Group B; *n* = 45) received three sessions per week of treatment as usual, which involved applied behavioural analysis (ABA) and intensive behavioural intervention (IBI) methods. The treatment group were allowed access to these ABA/IBI methods where necessary; however, behaviour interventions were not the focus within the treatment group given that the TF‐CBT protocol was used. Participants were then rated on levels of clinical symptoms (somatic; withdrawn; social problems; anxious/depressed; thought problems; attention problems; aggressive behaviour and rule breaking) using the Teacher Report Form of the Achenbach System of Empirically Based Assessment (ASEBA; Achenbach, 2001). Teachers were blind to which group participants had been allocated.

Results showed that in Group B, there was a significant decrease in all ASEBA subscales with the exception of the somatic subscale from pre‐ to post‐intervention. Thus, the control participants showed decreased symptomology in seven subscales after the intervention. However, less change was observed in Group A. The only subscale that showed a significant decrease was the aggressive behaviour subscale. The authors argued that TF‐CBT in conjunction with limited ABA/IBI was not as effective as ABA/IBI services for children with complex trauma and cognitive disabilities. They argued that this might be a result of the additional challenges disabilities pose in treating trauma as well as children having difficulty understanding the TF‐CBT tasks.

Haydon and Kennedy‐Donica (2023) presented a case study of a student with a learning disability who presented with academic, behavioural, and mental health needs as well as past sexual abuse trauma. The student was referred for mental health services by a teacher after engaging in self‐harm behaviour. The student underwent ACT, which involved a 12‐h on‐demand course and a one‐day in‐person follow‐up training. In addition, the student attended several shorter training sessions. Check‐in/out procedures were also used. Results suggested that intervention methods improved the student's processing of traumatic distress and emotion regulation. It was argued that these skills enabled the student to achieve personal goals.

### What are the barriers and facilitators to the implementation of TIPs designed to support CYP with ID?

The studies included in the current review provide valuable insights into the factors that act as barriers and facilitators when implementing TIPs with this population. These are presented below.

#### Barriers

Lack of resources (Chudzik, Corr, & Fisher, 2023) and lack of training (Chudzik, Corr, & Santos, 2023) impact upon the ability of teachers to implement trauma‐informed care. Relatedly, Schofield et al. (2021) argued that a lack of understanding of the young person's needs is another important barrier. An example of this comes from Chudzik, Corr, and Santos (2023) who argued that teachers may find it difficult to determine if symptoms are a result of the disability or from the traumatic event. More resources and better training may enhance teachers' awareness of their students' needs.

Another suggested barrier concerned the lack of additional staff members in schools such as paraprofessionals or classroom assistants, which resulted in teachers receiving minimal assistance throughout the day (Chudzik, Corr, & Fisher, 2023). It should be noted, though, that Goldenthal et al. (2024) argued that paraprofessionals may be a potential barrier to TIP given their lack of trauma training. Another issue related to staffing, identified by Chudzik, Corr, and Fisher (2023) and Goldenthal et al. (2024), was high turnover among staff members. It was argued that this resulted in newer members of staff missing training that helped to address trauma. Schools did not always have funds to implement the training again. Chudzik, Corr, and Santos (2023) also argued that the frequency with which classroom teacher groups were re‐arranged acted as a barrier, as this impacted upon consistency, routine, and trust building experienced by the student. Staffing challenges also came from limited mental health trained professionals within schools and the challenges associated with identifying counselling providers outside of school qualified to work with CYP with disabilities. There is a need for additional support from external mental health providers (Chudzik, Corr, & Santos, 2023).

Finally, differing views among professionals were also identified as a barrier to TIP. Chudzik, Corr, and Fisher (2023) argued that teachers found it challenging when there were inconsistent views within their schools about appropriate trauma‐informed strategies. Further, Goldenthal et al. (2024) argued that education authorities/districts have different practices and procedures placing importance on different educational strategies. This inconsistency across areas can act as a barrier to the implementation of appropriate TIPs.

#### Facilitators

Echoing the findings from barriers to successful implementation of TIPs with CYP with intellectual disability was the argument that a facilitator was teacher training. Several papers reported that effective TIP is achieved through continuous and embedded training for teachers and the need for trauma training should be written into educational policy (Chudzik, Corr, & Fisher, 2023; D'Amico et al., 2021; Holstead & Dalton, 2013; Opoku et al., 2024). Teachers must have in‐depth knowledge of trauma and possess the skills needed to support students with intellectual disability who have experienced trauma. Buy in from staff (Chudzik, Corr, & Santos, 2023), teacher experience (D'Amico et al., 2021) and providing teachers with time and space for reflection on their own learning and practice (Southall, 2024) were also reported as important. Opoku et al. (2024) argued that the provision of training programmes is the starting point for preparing teachers to better manage trauma in their classrooms.

In addition, it was argued that adaptations to usual trauma therapy sessions can facilitate treatment effectiveness. For example, reducing the length and pace of sessions, adding additional sessions, increasing the frequency of child and/or caregiver sessions, and adjustment of treatment content were seen as appropriate strategies (D'Amico et al., 2021; Goldenthal et al., 2024; Gray et al., 2024; Holstead & Dalton, 2013; Opoku et al., 2024). Within these papers, it was frequently suggested that treatment should take place over a longer period. The number of overall sessions should increase, but each individual session should be shorter and delivered at a slower pace using communication and visual aids. More caregiver involvement was also encouraged.

### Quality assessment

Using the MMAT, we were able to confirm that most of the papers included in the review answered the research questions set, used appropriate data collection and analysis methods, and made interpretations that were substantiated by data. As such, it was deemed that no paper included was of low quality, but that some sources of bias were present. More specifically, 6 of the 11 articles eligible for review received positive ratings for all questions, three studies had one negative rating and two studies had two negative ratings. A potential source of bias within the qualitative studies was research questions not being clearly stated (*n* = 1). Sources of potential bias within the quantitative studies related to the representativeness of the study samples (*n* = 1) and if confounders were accounted for in the design and analysis (*n* = 1). Potential sources of bias within the mixed methods studies were the lack of justification around the use of mixed methods and discussion of discrepancies/similarities within the quantitative and qualitative data (*n* = 2).

## DISCUSSION

The current study aimed to systematically evaluate, critically appraise and synthesize the available literature investigating TIPs in the context of working with CYP with intellectual disability. The review examined what TIPs, policies and models have been implemented, the effectiveness of such practices and possible barriers and facilitators in TIPs for this population. Such a review is important in enhancing knowledge of TIP within this population, what interventions are successful and factors that may benefit or hinder success. Key findings are now discussed.

The review identified several TIPs that have been reported within the context of supporting CYP with intellectual disability. SAMHSA, TF‐CBT, ACT, check in/check out, a tiered approach; a trauma‐informed resilience building curriculum; teacher training on attachment theory and the impact of fear on children's brain development were discussed. Some practices focused on whole‐school changes. For example, SAMHSA (2014) trauma‐informed care framework aims to create schools that endorse practices and policies that are sensitive to the support needs of those who have experienced trauma. Similarly, in Tier I of Goldenthals et al. (2024) tiered approach, the aim is to transform the school climate and use school‐wide strategies to address teaching and learning, while Tier II involves access to and implementation of school‐based behavioural intervention. Further, Southall's (2024) focus is on school staff training, which also relates to changes within the school climate.

Other papers focused more on skill building within the CYP. For example, D'Amico et al. (2021) and Holstead & Dalton (2013) discussed TF‐CBT, which aims to address negative thinking patterns around self‐blame, safety and dependency on others. It is hoped that the individual will develop coping skills and ways to restructure maladaptive thoughts. Similar to TF‐CBT, Gray et al. (2024) discussion of a resilience building curriculum aimed to teach the CYP how to cope with and minimize the risk of trauma exposure. Interestingly, although Haydon and Kennedy‐Donica's (2023) focus was also on the CYP who had experienced trauma, they discussed ACT, which works differently to TF‐CBT in that the individual is encouraged not to change their thinking patterns but to accept one's thoughts. It is important to note that Haydon and Kennedy‐Donica (2023) found support for ACT in an intervention case study.

It should be noted though that even when authors cited the same model, there was variation in how this was implemented. For example, studies utilizing SAMHSA (2014) drew from different parts of the framework: some drew on the principles, some on the areas of application and some on the 4R model (see Chudzik, Corr, & Fisher, 2023; Chudzik, Corr, & Santos, 2023; Chudzik, Corr, & Wolowiec‐Fisher, 2023; Opoku et al., 2024; Schofield et al., 2021). Similarly, despite the same overall aim of TF‐CBT within the D'Amico et al. (2021) and Holstead and Dalton (2013), both papers' descriptions of TF‐CBT were different. As the same theoretical framework is operationalized differently across studies, this raises questions about intervention fidelity which requires further exploration. In Holstead and Dalton's (2013) intervention study, it was reported that ABA/IBI was more effective than TF‐CBT. It was argued that this may be a result of the complex challenges CYP with intellectual disability and who have experienced trauma face as well as difficulty understanding the TF‐CBT tasks. It is now important to understand if such findings generalize across TF‐CBT or were specific to the intervention designed by Holstead and Dalton (2013).

It is problematic that the review found limited intervention studies in this area. Most studies focused on understanding teachers' attitudes towards trauma, their experiences of implementing TIP and examining their professional development needs. There is a need for research to examine the impact of (1) different models; and (2) different implementations of the same model upon intervention effectiveness as well as the impact upon the experiences of those involved in providing or receiving the intervention. Such research would assist in determining what interventions are most suitable for this population and how best these are implemented.

The review also identified barriers and facilitators to the implementation of TIPs designed to support CYP with intellectual disability. A lack of staff training was commonly reported as problematic (Chudzik, Corr, & Fisher, 2023; Goldenthal et al., 2024; Schofield et al., 2021). It was argued that classroom teachers and paraprofessionals may not receive adequate trauma training, which impacts on their ability to support those who have experienced trauma. This is not helped by high staff turnover and frequent changes in how classrooms are staffed (Chudzik, Corr, & Santos, 2023; Goldenthal et al., 2024). Teacher training was also discussed as a facilitator. It was argued that continuous and embedded trauma training for teachers should be written into educational policy (Chudzik, Corr, & Santos, 2023; D'Amico et al., 2021; Holstead & Dalton, 2013; Opoku et al., 2024). A barrier in accessing such training may relate to education districts supporting different practices and policies (Chudzik, Corr, & Santos, 2023; Goldenthal et al., 2024). Our review suggests the need for decision‐makers to implement policy around trauma training for teachers. It is important to note that teacher training must help teachers support students psychologically as well as support their learning. Given that another commonly stated facilitator was adaptations to usual trauma therapy sessions (D'Amico et al., 2021; Goldenthal et al., 2024; Gray et al., 2024; Holstead & Dalton, 2013; Opoku et al., 2024), such training should provide staff with knowledge to tailor trauma‐informed interventions to the individual needs of the CYP with intellectual disability that they support.

### Implications

The review findings have implications for research, practice, and policy. In relation to research, there is a need for studies to determine which model offers the best approach to supporting CYP with intellectual disability who have experienced trauma. Further, where a model or intervention has been used in different ways, the optimal way to implement this should be further investigated. Such work will provide clearer guidelines on the efficacy of specific interventions and how these should be delivered. More work is also needed around the effectiveness of trauma‐informed interventions for CYP with intellectual disability, given that one study did not find this to work with this population. More intervention evaluation research is needed across this field. Our review also showed that most research focused on the experiences of teachers, special education professionals, clinical staff and school social workers who had used TIP for CYP with intellectual disability and thus cannot fully address intervention effectiveness. Thus, an important gap identified by the review is that most research focused on professional experiences rather than those of CYP with intellectual disability. This represents a significant limitation that reflects broader issues in disability research about whose voices are centred in knowledge production (e.g., Mietola et al., 2017). More intervention evaluation research is needed which empowers CYP with intellectual disability to share their experiences of TIP. Further, our review did not identify experiences unique to each type of education and clinical practitioner included within the studies. While this suggests that professionals working with the field of TIP for CYP with intellectual disability hold similar views, future research should take this into account as it may be important to compare their experiences and attitudes.

Our findings have provided insights into barriers and facilitators of TIP implementation for CYP with ID. Researchers may now wish to consider these within implementation science frameworks. This would allow for an in‐depth understanding of why certain TIPs succeed or fail in different contexts by examining how they interact and influence implementation outcomes.

In relation to practice, it is important to acknowledge the finding that only three studies examined intervention effectiveness, with one study (Holstead & Dalton, 2013) finding limited effectiveness of TF‐CBT compared to standard behavioural approaches. This has implications for evidence‐based practice. It should be noted, though, that more research is needed to further examine this, given the limited number of studies in this area.

We also encourage those involved in developing and delivering trauma‐informed interventions to CYP with intellectual disability to make adaptations to usual trauma therapy sessions. This involves adapting the timing, frequency and content of sessions as well as providing more caregiver involvement. We also argue for more opportunities for collaboration between school staff and externally trained professionals such as mental health practitioners or counsellors given that they will be able to provide support to schools. Our findings also suggest the need for national and international education policy to prioritize teacher training that is embedded and continuous for school staff. Teacher training must help teachers support students psychologically as well as academically.

### Strengths and limitations

The review has several strengths. For example, we used PRISMA‐ScR guidelines to ensure we carried out a comprehensive literature search. We also implemented reliability checks and studies were evaluated against methodological criteria. This meant we were able to consider quality of evidence. However, the review has possible limitations which should be acknowledged. For example, only peer‐reviewed articles written in English language were included. Although there are advantages to this approach, it should be noted that this may create a bias towards studies that have been published, and more likely then, their hypotheses supported. In addition to this, our search strategy may be viewed as constrained as we focused on the terms ‘intellectual disability’ and ‘learning disability’ only rather than including all diagnostic labels associated with intellectual disability. Despite this, our review includes papers recruiting a range of populations such as intellectual disability, learning disability, developmental disabilities, developmental delay, cognitive disabilities and Prader‐Willi Syndrome giving us confidence in our search terms. It should be noted that 9 out of the 11 papers included in the review were based in the United States. Such a finding highlights the limited research attention TIP in CYP with intellectual disability has been given in other countries and thus an important area for future research. It may therefore be difficult to generalize our findings to countries outside of the United States given differences in health care, education and social service systems internationally. The generalisability of findings across different cultural and systemic contexts requires more explicit consideration, particularly given the varying approaches to both TIP and intellectual disability support across different countries. Finally, it should be noted that the quality assessment process highlighted that some included studies had methodological limitations that may impact the reliability of their findings.

## CONCLUSION

This is the first scoping review to examine TIPs in the context of working with CYP with intellectual disability. The review identified several trauma‐informed models and practices that have been reported within the context of supporting CYP with intellectual disability. Some studies focused on whole‐school changes which aimed to transform the school climate by implementing trauma‐informed teaching and learning strategies. Other studies focused on skill building within CYP through therapeutic intervention. Despite this, our findings indicated that there are limited intervention studies in this context, and this is an important area of future research. Barriers and facilitators in the implementation of TIPs were also identified and these have important implications for decision‐makers around international education practice and policy. We urge education practitioners to make adaptations to usual trauma therapy sessions when working with CYP with intellectual disability and to seek collaboration from mental health experts. Further, policymakers must recognize the importance of teacher training around trauma for this population and take action to provide such opportunities to school staff. In doing so, staff will become empowered to help CYP with intellectual disability to overcome trauma‐related barriers and achieve more positive developmental outcomes.

## AUTHOR CONTRIBUTIONS

**Claire Wilson:** Conceptualization; investigation; writing – original draft; writing – review and editing; methodology; project administration. **Zara P. Brodie:** Conceptualization; investigation; writing – original draft; writing – review and editing; methodology. **Kirsten Russell:** Conceptualization; investigation; writing – original draft; methodology; writing – review and editing; project administration.

## CONFLICT OF INTEREST STATEMENT

The authors declare no conflicts of interest.

## Data Availability

Data sharing is not applicable to this article as no new data were created or analyzed in this study.

## APPENDIX A

### A.1 Search terms

Our search terms are: (‘trauma informed’ OR ‘trauma focused’ OR ‘trauma responsive’ OR ‘trauma‐informed’ OR ‘trauma‐focused’ OR ‘trauma‐responsive’) AND (intellectual disabilit * OR learning disabilit* OR learning difficult* OR intellectual development disorder OR intellectual impairment OR mental retardation).

Please note that although ‘mental retardation’ is now an outdated term, we included this to avoid missing studies published when this term was acceptable.

## References

[bjep70011-bib-0071] Achenbach, T. M. (2001). Manual for the ASEBA school‐age forms & profiles. University of Vermont.

[bjep70011-bib-0072] Allen, B. , & Johnson, J. C. (2012). Utilization and implementation of trauma‐focused cognitive–behavioral therapy for the treatment of maltreated children. Child maltreatment, 17, 80–85. 10.1177/1077559511418220 21875905

[bjep70011-bib-0001] American Psychiatric Association . (2013). Diagnostic and statistical manual of mental disorders (5th ed.). American Psychiatric Publishing.

[bjep70011-bib-0073] Arrieta, J. , Aguerrebere, M. , Raviola, G. , Flores, H. , Elliott, P. , & Mukherjee, J. (2017). Validity and utility of the patient health questionnaire (PHQ)‐2 and PHQ‐9 for screening and diagnosis of depression in rural Chiapas, Mexico: A cross‐sectional study. Journal of Clinical Psychology, 73, 1076–1090. 10.1002/jclp.22390 28195649 PMC5573982

[bjep70011-bib-0074] Baker, C. N. , Brown, S. M. , Wilcox, P. D. , Overstreet, S. , & Arora, P. (2016). Development and psychometric evaluation of the attitudes related to trauma‐informed care (ARTIC) scale. School Mental Health, 8, 61–76. 10.1007/s12310-015-9161-0

[bjep70011-bib-0002] Bath, H. , & Seita, J. (2008). The Three Pillars of Transforming care: Healing in the ‘other 23 hours’. The article updates and expands on articles in Reclaiming Children and Youth, 1–13.

[bjep70011-bib-0003] Bellis, M. A. , Hughes, K. , Leckenby, N. , Hardcastle, K. A. , Perkins, C. , & Lowey, H. (2015). Measuring mortality and the burden of adult disease associated with adverse childhood experiences in England: A national survey. Journal of Public Health, 37, 445–454. 10.1093/pubmed/fdu065 25174044 PMC4552010

[bjep70011-bib-0004] Bowlby, J. (1988). A secure base: Parent‐child attachment and healthy human development. Basic Books.

[bjep70011-bib-0005] Bronfenbrenner, U. (1977). Toward an experimental ecology of human development. American Psychologist, 32, 513–531.

[bjep70011-bib-0006] Cai, J. , Li, J. , Liu, D. , Gao, S. , Zhao, Y. , Zhang, J. , & Liu, Q. (2023). Long‐term effects of childhood trauma subtypes on adult brain function. Brain and Behavior: A Cognitive Neuroscience Perspective, 13, e2981. 10.1002/brb3.2981 PMC10175996 36974448

[bjep70011-bib-0007] Chudzik, M. , Corr, C. , & Fisher, K. W. (2023). Trauma‐informed care: The professional development needs of early childhood special education teachers. Journal of Early Intervention, 46, 113–129. 10.1177/10538151231164898

[bjep70011-bib-0008] Chudzik, M. , Corr, C. , & Santos, R. M. (2023). “… We're not doing enough:”: Trauma‐informed Care in an Early Childhood Special Education Center. Topics in Early Childhood Special Education, 45, 02711214231219282.

[bjep70011-bib-0009] Chudzik, M. , Corr, C. , & Wolowiec‐Fisher, K. (2023). Trauma: Early childhood special education teachers' attitudes and experiences. Early Childhood Education Journal, 51, 189–200. 10.1007/s10643-021-01302-1

[bjep70011-bib-0010] Cohen, J. A. , Mannarino, A. P. , & Deblinger, E. (2006). Treating trauma and traumatic grief in children and adolescents. Guilford.

[bjep70011-bib-0011] Cohen, J. A. , Mannarino, A. P. , & Deblinger, E. (2017). Treating trauma and traumatic grief in children and adolescents (2nd ed.). Guilford Press.

[bjep70011-bib-0075] Cook, A. , Spinazzola, J. , Ford, J. , Lanktree, C. , Blaustein, M. , Cloitre, M. , & Van der Kolk, B. (2005). Complex trauma. Psychiatric Annals, 35, 390–398.

[bjep70011-bib-0012] Cook, S. , & Hole, R. (2021). Trauma, intellectual and/or developmental disability, and multiple, complex needs: A scoping review of the literature. Research in Developmental Disabilities, 115, 103939. 10.1016/j.ridd.2021.103939 33934926

[bjep70011-bib-0076] Craig, S. E. (2016). Trauma‐sensitive schools: Learning communities transforming children's lives, K5. Teachers College Press.

[bjep70011-bib-0013] D'Amico, P. J. , Vogel, J. M. , Mannarino, A. P. , Hoffman, D. L. , Briggs, E. C. , Tunno, A. M. , & Schwartz, R. M. (2021). Tailoring trauma‐focused cognitive behavioral therapy (TF‐CBT) for youth with intellectual and developmental disabilities: A survey of nationally certified TF‐ CBT therapists. Evidence‐Based Practice in Child and Adolescent Mental Health, 7, 112–124. 10.1080/23794925.2021.1955639

[bjep70011-bib-0014] Dion, J. , Paquette, G. , Tremblay, K. N. , Collin‐Vesina, D. , & Chabot, M. (2018). Child maltreatment among children with intellectual disability in the Canadian incidence study. American Journal of Intellectual and Developmental Disabilities, 123, 176–188. 10.1352/1944-7558-123.2.176 29480775

[bjep70011-bib-0015] Emerson, E. , Baines, S. , & Allerton, L. (2011). Health inequalities and people with learning disabilities in the UK. Tizard Learning Disability Review, 8, 16–42. 10.5042/tldr.2011.0008

[bjep70011-bib-0016] Felitti, V. J. , Anda, R. F. , Nordenberg, D. , Williamson, D. F. , Spitz, A. M. , Edwards, V. , & Marks, J. S. (1998). Relationship of childhood abuse and household dysfunction to many of the leading causes of death in adults: The adverse childhood experiences (ACE) study. American Journal of Preventive Medicine, 14, 245–258. 10.1016/s0749-3797(98)00017-8 9635069

[bjep70011-bib-0077] Foa, E. B. , Johnson, K. M. , Feeny, N. C. , & Treadwell, K. R. (2001). The child PTSD symptom scale: A preliminary examination of its psychometric properties. Journal of Clinical Child & Adolescent Psychology, 30, 376–384. 10.1207/S15374424JCCP3003_9 11501254

[bjep70011-bib-0017] Follette, V. M. , Polusny, M. A. , Bechtle, A. E. , & Naugle, A. E. (1996). Cumulative trauma: The impact of child sexual abuse, adult sexual assault, and spouse abuse. Journal of Traumatic Stress, 9, 25–35. 10.1002/jts.2490090104 8750449

[bjep70011-bib-0018] Gardani, M. , Bradford, D. R. , Russell, K. , Allan, S. , Beattie, L. , Ellis, J. G. , & Akram, U. (2022). A systematic review and meta‐analysis of poor sleep, insomnia symptoms and stress in undergraduate students. Sleep Medicine Reviews, 61, 101565. 10.1016/j.smrv.2021.101565 34922108

[bjep70011-bib-0078] Goldenthal, H. J. , Gill, T. , Rivera, C. , Gouze, K. R. , & Cicchetti, C. (2024). Implementing trauma‐informed care in a special education setting: An initial exploration of a multi‐tiered model. Evaluation and Program Planning, 103, 102407. 10.1016/j.evalprogplan.2024.102407 38367349

[bjep70011-bib-0019] Gray, K. , Marlotte, L. , Aralis, H. , Kaufman, J. , Kataoka, S. , Venegas‐Murillo, A. , Lester, P. , Escudero, P. , & Ijadi‐Maghsoodi, R. (2024). Understanding and addressing the needs of students in special education through a trauma‐informed resilience curriculum. Social Work in Public Health, 39, 405–421. 10.1080/19371918.2024.2316866 38722275 PMC11530001

[bjep70011-bib-0079] Harris, M. , & Fallot, R. D. (2006). Envisioning a trauma‐informed service system: A vital paradigm shift. New Directions for Mental Health Services, 2001, 3–22. 10.1002/yd.23320018903 11291260

[bjep70011-bib-0080] Hart, C. M. , Ritchie, T. D. , Hepper, E. G. , & Gebauer, J. E. (2015). The balanced inventory of desirable responding short form (BIDR‐16). SAGE Open, 5, 2158244015621113. 10.1177/2158244015621113

[bjep70011-bib-0020] Hatton, C. , & Emerson, E. (2015). Introduction: Health disparities, health inequity, and people with intellectual disabilities. In International review of research in developmental disabilities. Elsevier.

[bjep70011-bib-0021] Hawken, L. S. , Crone, D. A. , Bundock, K. , & Horner, R. H. (2020). Responding to problem behavior in schools: The behavior education program (3rd ed.). Guilford.

[bjep70011-bib-0022] Haydon, T. , & Kennedy‐Donica, A. (2023). Implementing acceptance and commitment therapy and check‐in/check‐out with a struggling learner: A case report. Insights into Learning Disabilities, 20, 81–99.

[bjep70011-bib-0023] Hayes, S. C. (2016). Acceptance and commitment therapy, relational frame theory, and the third wave of behavioural and cognitive therapies – Republished article. Behavior Therapy, 47, 869–885. 10.1016/S0005-7894(04)80013-3 27993338

[bjep70011-bib-0024] Holstead, J. , & Dalton, J. (2013). Utilization of trauma‐focused cognitive behavioral therapy (TF‐CBT) for children with cognitive disabilities. Journal of Public Child Welfare, 7, 536–548. 10.1080/15548732.2013.843495

[bjep70011-bib-0025] Hong, Q. N. , Fàbregues, S. , Bartlett, G. , Boardman, F. , Cargo, M. , Dagenais, P. , Gagnon, M.‐P. , Griffiths, F. , Nicolau, B. , O'Cathain, A. , Rousseau, M.‐C. , Vedel, I. , & Pluye, P. (2018). The mixed methods appraisal tool (MMAT) version 2018 for information professionals and researchers. Education for Information, 34, 285–291. 10.3233/EFI-180221

[bjep70011-bib-0026] Hughes, D. A. , Golding, K. S. , & Hudson, J. (2019). Healing relational trauma with attachment‐focused interventions: Dyadic developmental psychotherapy with children and families. WW Norton & Company.

[bjep70011-bib-0027] Hurt, H. , Malmud, E. , Brodsky, N. L. , & Giannetta, J. (2001). Exposure to violence: Psychological and academic correlates in child witnesses. Archives of Pediatrics & Adolescent Medicine, 155, 1351–1356. 10.1001/archpedi.155.12.1351 11732955

[bjep70011-bib-0028] Jones, L. , Bellis, M. A. , Wood, S. , Hughes, K. , McCoy, E. , Eckley, L. , Bates, G. , Mikton, C. , Shakespeare, T. , & Officer, A. (2012). Prevalence and risk of violence against children with disabilities: A systematic review and meta‐analysis of observational studies. The Lancet, 380, 899–907. 10.1016/S0140-6736(12)60692-8 22795511

[bjep70011-bib-0029] Kerns, C. M. , Newschaffer, C. J. , & Berkowitz, S. J. (2015). Traumatic childhood events and autism spectrum disorder. Journal of Autism and Developmental Disorders, 45, 3475–3486. 10.1007/s10803-015-2392-y 25711547

[bjep70011-bib-0030] Kildahl, A. N. , Bakken, T. L. , Iversen, T. E. , & Helverschou, S. B. (2019). Identification of post‐traumatic stress disorder in individuals with autism spectrum disorder and intellectual disability: A systematic review. Journal of Mental Health Research in Intellectual and Developmental Disabilities, 12(1‐2), 1–25. 10.1080/19315864.2019.1595233

[bjep70011-bib-0031] Kildahl, A. N. , Helverschou, S. B. , & Oddli, H. W. (2019). Clinicians' retrospective perceptions of failure to detect sexual abuse in a young man with autism and mild intellectual disability. Journal of Intellectual & Developmental Disability, 45, 194–202. 10.3109/13668250.2019.1680821

[bjep70011-bib-0081] Knight, J. R. , Sherritt, L. , Shrier, L. A. , Harris, S. K. , & Chang, G. (2002). Validity of the CRAFFT substance abuse screening test among adolescent clinic patients. Archives of Pediatrics & Adolescent Medicine, 156, 607–614. 10.1001/archpedi.156.6.607 12038895

[bjep70011-bib-0032] Ko, S. J. , Ford, J. D. , Kassam‐Adams, N. , Berkowitz, S. J. , Wilson, C. , Wong, M. , Brymer, M. J. , & Layne, C. M. (2008). Creating trauma‐informed systems: Child welfare, education, first responders, health care, juvenile justice. Professional Psychology: Research and Practice, 39, 396–404. 10.1037/0735-7028.39.4.396

[bjep70011-bib-0033] Landis, J. R. , & Gary, G. K. (1977). The measurement of observer agreement for categorical data. Biometrics, 33, 159–174. 10.2307/2529310 843571

[bjep70011-bib-0034] Lee, K. , Cascella, M. , & Marwaha, R. (2023). Intellectual disability. In StatPearls. StatPearls Publishing. 31613434

[bjep70011-bib-0082] Lisitski, J. L. (2019). The use of trauma‐informed care in programs serving families experiencing homelessness. (Doctoral dissertation, Bryn Mawr College, Graduate School of Social Work and Social Research).

[bjep70011-bib-0035] Luciano, C. , Valdivia‐Salas, S. , & Ruiz, F. J. (2012). The self as the context for rule‐governed behavior. In L. McHugh & I. Stewart (Eds.), The self and perspective taking: Research and applications (pp. 143–160). Context Press.

[bjep70011-bib-0036] Luke, N. , & Coyne, S. M. (2008). Fostering self‐esteem: Exploring adult recollections on the influence of foster parents. Child & Family Social Work, 13, 402–410. 10.1111/j.1365-2206.2008.00565.x

[bjep70011-bib-0037] Mason‐Roberts, S. , Bradley, A. , Karatzias, T. , Brown, M. , Paterson, D. , Walley, R. , Truesdale, M. , Taggart, L. , & Sirisena, C. (2018). Multiple traumatisation and subsequent psychopathology in people with intellectual disabilities and DSM‐5 PTSD: A preliminary study. Journal of Intellectual Disability Research, 62, 730–736. 10.1111/jir.12505 29856097

[bjep70011-bib-0038] McDonnell, C. G. , Boan, A. D. , Bradley, C. C. , Seay, K. D. , Charles, J. M. , & Carpenter, L. A. (2019). Child maltreatment in autism spectrum disorder and intellectual disability: Results from a population‐based sample. Journal of Child Psychology and Psychiatry, 60, 576–584. 10.1111/jcpp.12993 30368827 PMC6458088

[bjep70011-bib-0039] McGlivery, S. (2018). The identification and treatment of trauma in individuals with developmental disabilities. NADD Press.

[bjep70011-bib-0083] McInerney, M. , & McKlindon, A. (2015). Unlocking the door to learning: Trauma‐informed classrooms and transformational schools. Education Law Center.

[bjep70011-bib-0040] McNally, P. , Taggart, L. , & Shevlin, M. (2021). Trauma experiences of people with an intellectual disability and their implications: A scoping review. Journal of Applied Research in Intellectual Disabilities, 34, 927–949. 10.1111/jar.12872 33772975

[bjep70011-bib-0041] Mencap . (2024). What is a learning disability? http://www.mencap.org.uk/definition

[bjep70011-bib-0042] Mevissen, L. , & de Jongh, A. (2010). PTSD and its treatment in people with intellectual disabilities: A review of the literature. Clinical Psychology Review, 30, 308–316. 10.1016/j.cpr.2009.12.005 20056303

[bjep70011-bib-0043] Mevissen, L. , Didden, R. , & de Jongh, A. (2016). Assessment and treatment of PTSD in people with intellectual disabilities. In C. R. Martin , V. R. Preedy , & V. B. Patel (Eds.), Comprehensive guide to post‐traumatic stress disorder (1st ed., pp. 281–299). Springer.

[bjep70011-bib-0044] Mietola, R. , Miettinen, S. , & Vehmas, S. (2017). Voiceless subjects? Research ethics and persons with profound intellectual disabilities. International Journal of Social Research Methodology, 20, 263–274.

[bjep70011-bib-0045] Morgan, A. , Pendergast, D. , Brown, R. , & Heck, D. (2015). Relational ways of being an educator: Trauma‐informed practice supporting disenfranchised young people. International Journal of Inclusive Education, 19, 1037–1051. 10.1080/13603116.2015.1035344

[bjep70011-bib-0046] Nouwens, P. J. , Lucas, R. , Smulders, N. B. , & Embregts, P. J. (2017). Identifying classes of persons with mild intellectual disability or borderline intellectual functioning: A latent class analysis. BMC Psychiatry, 2017(17), 1–9. 10.1186/s12888-017-1426-8 PMC5512980 28716016

[bjep70011-bib-0047] Opoku, M. P. , Elhoweris, H. , Moustafa, A. , Miezah, D. , Shah, H. , & Oppong, A. (2024). Perceived competence of teachers in the implementation of trauma‐informed practices for students with disabilities in classrooms in The United Arab Emirates. Journal of Child & Adolescent Trauma, 17, 611–625. 10.1007/s40653-023-00591-5 38938937 PMC11199469

[bjep70011-bib-0084] Perry, B. D. (2006). Applying principles of neurodevelopment to clinical work with maltreated and traumatized children: The neurosequential model of therapeutics. In N. B. Webb (Ed.), Working with traumatized youth in child welfare (pp. 27–52). The Guilford Press.

[bjep70011-bib-0048] Reiter, S. , Bryen, D. N. , & Shachar, I. (2007). Adolescents with intellectual disabilities as victims of abuse. Journal of Intellectual Disabilities, 11, 371–387. 10.1177/1744629507084602 18029413

[bjep70011-bib-0049] Rittmannsberger, D. , Kocman, A. , Weber, G. , & Lueger‐Schuster, B. (2018). Trauma exposure and post‐traumatic stress disorder in people with intellectual disabilities: A Delphi expert rating. Journal of Applied Research in Intellectual Disabilities, 32, 558–567. 10.1111/jar.12549 30453387

[bjep70011-bib-0050] Russell, K. , Allan, S. , Beattie, L. , Bohan, J. , MacMahon, K. , & Rasmussen, S. (2019). Sleep problem, suicide and self‐harm in university students: A systematic review. Sleep Medicine Reviews, 44, 58–69. 10.1016/j.smrv.2018.12.008 30721844

[bjep70011-bib-0051] Sacchi, L. , Merzhvynska, M. , & Augsburger, M. (2020). Effects of cumulative trauma load on long‐term trajectories of life satisfaction and health in a population‐based study. BMC Public Health, 20, 1612. 10.1186/s12889-020-09663-9 33109171 PMC7590721

[bjep70011-bib-0052] Schofield, C. , Martin, K. , Choong, C. S. , Gibson, D. , Skoss, R. , & Downs, J. (2021). Using a trauma informed practice framework to enhance understanding of and identify support strategies for behavioural difficulties in young people with Prader‐Willi syndrome. Research in Developmental Disabilities, 110, 103839. 10.1016/j.ridd.2020.103839 33482559

[bjep70011-bib-0053] Scotti, J. R. , Stevens, S. B. , Jacoby, V. M. , Bracken, M. R. , Freed, R. , & Schmidt, E. (2012). Trauma in people with intellectual and develop‐ mental disabilities: Reactions of parents and caregivers to research participation. Intellectual and Developmental Disabilities, 50, 199–206. 10.1352/1934-9556-50.3.199 22731969

[bjep70011-bib-0054] Skelly, A. (2020). Trauma exposure and the importance of attachment in people with intellectual disabilities. FPID Bulletin: The Bulletin of the Faculty for People with Intellectual Disabilities, 18, 15–19.

[bjep70011-bib-0055] Snowman, J. , & McCown, R. (2012). Ed Psych. Cengage Learning.

[bjep70011-bib-0056] Souers, K. , & Hall, P. (2016). Fostering resilient learners: Strategies for creating a trauma‐sensitive classroom. ASCD.

[bjep70011-bib-0057] Southall, A. (2024). The trauma challenge: How teachers experience students with complex trauma. British Journal of Special Education, 51, 3–14. 10.1111/1467-8578.12487

[bjep70011-bib-0058] Soylu, N. , Alpaslan, A. H. , Ayaz, M. , Esenyel, S. , & Oruc, M. (2013). Psychiatric disorders and characteristics of abuse in sexually abused children and adolescents with and with‐out intellectual disabilities. Research in Developmental Disabilities, 34, 4334–4342. 10.1016/j.ridd.2013.09.010 24161460

[bjep70011-bib-0085] Spitzer, R. L. , Kroenke, K. , Williams, J. B. , & Löwe, B. (2006). A brief measure for assessing generalized anxiety disorder: The GAD‐7. Archives of Internal Medicine, 166, 1092–1097. 10.1001/archinte.166.10.1092 16717171

[bjep70011-bib-0086] Stamm, B. H. (2010). The concise ProQOL manual (2nd ed.). ProQOL.org.

[bjep70011-bib-0087] Steele, W. , & Malchiodi, C. A. (2012). Trauma‐informed practices with children and adolescents. Routledge/Taylor & Francis Group.

[bjep70011-bib-0059] Stewart‐Tufescu, A. , Struck, S. , Taillieu, T. , Salmon, S. , Fortier, J. , Brownell, M. , Chartier, M. , Yakubovich, A. R. , & Afifi, T. O. (2022). Adverse childhood experiences and education outcomes among adolescents: Linking survey and administrative data. International Journal of Environmental Research and Public Health, 19, 11564. 10.3390/ijerph191811564 36141833 PMC9517426

[bjep70011-bib-0060] Substance Abuse and Mental Health Services Administration . (2014). SAMHSA's concept of trauma and guidance for a trauma‐informed approach. https://library.samhsa.gov/sites/default/files/sma14‐4884.pdf

[bjep70011-bib-0061] Suliman, S. G. , Mkabile, D. S. , Fincham, R. A. , Stein, D. J. , & Seedat, S. (2009). Cumulative effect of multiple trauma on symptoms of posttraumatic stress disorder, anxiety, and depression in adolescents. Comprehensive Psychiatry, 50, 121–127. 10.1016/j.comppsych.2008.06.006 19216888

[bjep70011-bib-0062] Sullivan, P. M. , & Knutson, J. F. (2000). Maltreatment and disabilities: A population‐based epidemiological study. Child Abuse & Neglect, 24, 1257–1273. 10.1016/S0145-2134(00)00190-3 11075694

[bjep70011-bib-0063] The Complex Trauma Taskforce . (2012). The ISTSS expert consensus treatment guidelines for complex PTSD in adults. https://istss.org/ISTSS_Main/media/Documents/ISTSS‐Expert‐Concesnsus‐Guidelines‐for‐Complex‐PTSD‐Updated‐060315.pdf

[bjep70011-bib-0064] Tomasulo, D. J. , & Razza, N. J. (2007). Posttraumatic stress disorder. In R. Fletcher , E. Loschen , C. Stavrakaki , & M. First (Eds.), Diagnostic manual–intellectual disability (DM‐ID): A textbook of diagnosis of mental disorders in persons with intellectual disability (pp. 365–378). NADD Press.

[bjep70011-bib-0065] Tricco, A. C. , Lillie, E. , Zarin, W. , O'Brien, K. K. , Colquhoun, H. , Levac, D. , Moher, D. , Peters, M. D. J. , Horsley, T. , Weeks, L. , Hempel, S. , Akl, E. A. , Chang, C. , McGowan, J. , Stewart, L. , Hartling, L. , Aldcroft, A. , Wilson, M. G. , Garritty, C. , … Straus, S. E. (2018). PRISMA extension for scoping reviews (PRISMA‐ScR): Checklist and explanation. Annals of Internal Medicine, 169, 467–473. 30178033 10.7326/M18-0850

[bjep70011-bib-0066] Verdugo, M. A. , Aguayo, V. , Arias, V. B. , & García‐Domínguez, L. (2020). A systematic review of the assessment of support needs in people with intellectual and developmental disabilities. International Journal of Environmental Research and Public Health, 17, 9494. 10.3390/ijerph17249494 33352974 PMC7766556

[bjep70011-bib-0067] Vervoort‐Schel, J. , Mercera, G. , Wissink, I. , Van der Helm, P. , Lindauer, R. , & Moonen, X. (2021). Prevalence of and relationship between adverse childhood experiences and family context risk factors among children with intellectual disabilities and borderline intellectual functioning. Research in Developmental Disabilities, 113, 103935. 10.1016/j.ridd.2021.103935 33756254

[bjep70011-bib-0068] Wigham, S. , Hatto, C. , & Taylor, J. L. (2011). The effects of traumatising life events on people with intellectual disabilities: A systematic review. Journal of Mental Health Research in Intellectual Disabilities, 4, 19–39. 10.1080/19315864.2010.534576

[bjep70011-bib-0069] World Health Organization . (2001). International classification of functioning, disability, and health—ICF. Author.

[bjep70011-bib-0070] Zortea, T. C. , Gray, C. M. , & O'Connor, R. C. (2021). The relationship between adult attachment and suicidal thoughts and behaviors: A systematic review. Archives of Suicide Research, 25, 38–73. 10.1080/13811118.2019.1661893 31545148

